# In Vitro Effects of Propranolol on T Helper Type 1 Cytokine Profile in Human Leukemic T Cells

**Published:** 2016-04-01

**Authors:** Fatemeh Hajighasemi, Abbas Mirshafiey

**Affiliations:** 1 Department of Immunology, Faculty of Medicine, Shahed University, Tehran, Iran; 2 Department of Pathobiology, School of Public Health, Tehran University of Medical Sciences, Tehran, Iran

**Keywords:** Propranolol, Cytokines, Leukemic, T-cells

## Abstract

**Introduction:** Cytokines are a large group of proteins play a key role in inflammation. Down-regulation of pro-inflammatory cytokines has beneficial effect on heart function. Propranolol, as a non selective beta-adrenergic blocker, has been extensively used for treatment of many cardiovascular problems such as arrhythmias and heart malfunction. In addition anti-inflammatory effects of propranolol have been revealed. In this study the propranolol effect on T helper type 1 cytokine profile in human leukemic T cells has been assessed in vitro.

**Materials and methods:** Human leukemic T cells (Molt-4 and Jurkat) were cultured in complete RPMI medium. The cells were then incubated with different concentrations of propranolol (0.03- 30 µM) in the presence or absence of PHA (10 µg/ml) for 48 hours. The supernatants of cell culture media were collected and used for cytokines assay.

**Results:** Propranolol significantly decreased the T helper type 1 cytokine profile [Interleukin-2 (IL-2) and Interferon- γ (IFN-γ)] production in PHA stimulated Molt-4 and Jurkat cells, after 48 hour of incubation time, dose-dependently compared to untreated control cells.

**Conclusion:** Our data showed a dose dependent inhibitory effect of propranolol on the IL-2 and IFN-γ production in human leukemic Molt-4 and Jurkat cells. The anti- inflammatory effect of propranolol reported by other investigators may be in part due to its suppressive effect on production of inflammatory cytokines such as IL-2 and IFN-γ. So, propranolol along with its chronic long-term usage in cardiovascular problems may have potential implication in treatment of inflammatory-based disorders.

## Introduction

 Cytokines are a large group of proteins which are produced by a number of cells and play a key role in the immunopathogenesis of several disorders such as inflammatory bowel disease, periodontitis, Sjögren's syndrome and atherosclerosis.^1^^-^^4^ In addition deysregulation of cytokines in some patients has been shown.^5^^-^^7^ Modulation of cytokines has had promising results for treatment of some diseases such as leukemia and cardiac infarction.^8^^,^^9^ Improved heart function following down-regulation of proinflammatory cytokines has been reported.^10^^,^^11^ Propranolol, as a non selective beta-adrenergic blocker, has been extensively used for treatment of many cardiovascular problems such as ischemic heart diseases, arrhythmias and heart malfunction.^12^^,^^13^ In addition anti-inflammatory and antiangiogenesis effects of propranolol have been revealed.^14^^,^^15^ Moreover decrease of inflammatory cell and some cytokine (tumor necrosis factor-α and interleukin-8) levels in alveolar fluid of rat by propranolol has been reported in vivo.^16^ Besides the regulatory effect of propranolol on cytokine production in tumor-infiltrating lymphocytes and peripheral blood mononuclear cells (PBMCs) of colorectal cancer patients has been shown.^17^ In this study the propranolol effect on profile of T helper type 1 (Th1) cytokines in human T cell lines has been assessed in vitro.

## MATERIALS AND METHODS

**Materials**

RPMI-1640 medium, penicillin, streptomycin, phytoheamagglutinin (PHA), and trypan blue (TB) were from sigma (USA). Fetal calf serum (FCS) was from Gibco (USA). Interleukin-2 (IL-2) and Interferon- γ (IFN-γ) standard ELISA kit was obtained from R & D company (USA). Propranolol was a kind gift from HAKIM Pvt. Co. Ltd (Tehran, Iran). Microtiter plates, flasks and tubes were from Nunc (Falcon, USA).

**Cell lines**

Human leukemic T cells [Molt-4 (NCBI C149) and Jurkat (NCBI C121)], were obtained from NCBI (National Cell Bank of Iran, Pasteur Inst. of Iran, Tehran). The cells were maintained in RPMI-1640 medium supplemented with 10% FCS in 5% CO_2_ at 37°C.

**Preparation of propranolol**

Propranolol was dissolved in RPMI-1640 medium and stored at -20°C until use. Drug was diluted in culture medium to prepare the needed concentrations before use.

**Cell culture and treatment**

The method has been described in detail elsewhere.^15^ Briefly, the human leukemic cells were cultured in RPMI-1640 medium supplemented with 10% FCS, penicillin (100 IU/ml) and streptomycin (100 µg/ml) at 37°C in 5% CO_2_. The cells were seeded at a density of 2×10^6^ cell/ml and then incubated with different concentrations of propranolol (0.03- 30 µM) in the presence or absence of PHA (10 µg/ml) for 48 hours. The supernatants of cell culture media were collected and used for IL-2 and IFN-γ assay. All experiments were done in triplicate.

**Cytokines assay**

The amount of IL-2 and IFN-γ secreted in the cell culture supernatants by human leukemic T cell lines (Molt-4 and Jurkat) was measured with the Quantikine human enzyme-linked immunosorbent assay (ELISA) kits (R & D systems) according to the manufacturer’s instructions. This assay uses the quantitative sandwich enzyme immunoassay technique. Complete RPMI medium was used as control and human recombinant IL-2, and IFN-γ were employed as standard for drawing the standard curves.

**Statistical analysis**

Effect of the drug on each cell line was performed in three independent experiments and the results were expressed as mean ± SEM. Statistical comparisons among groups were made by analysis of variance (ANOVA). P<0.05 was considered significant. Test of multiple comparison of Tukey was applied (5%) for statistically significant differences. For statistical analysis and graph making, the software SPSS 11.5 and Excel 2013 were used, respectively.

## Results

**I)Propranolol effect on IL-2 production in human leukemic cell lines**

**Propranolol effect on IL-2 production in human Molt-4 cells**

**Figure 1:**
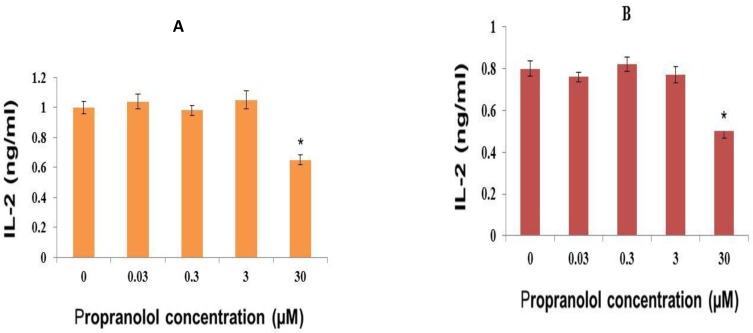
Effect on propranolol on IL-2 secretion by human leukemic (A) Molt-4 T-cell and (B) Jurkat T-cell lines. The cells (2 × 10 6 cells/ml) were treated with different concentrations of propranolol (0.03-30 μM) for 48 hours in the presence of PHA (10 µg/ml). At the end of treatment, IL-2 concentration in conditioned medium was measured by ELISA. Data are mean ± SEM of three independent experiments. *P<0.05 was considered significant.

IL-2 production was relatively low in unstimulated human leukemic Molt-4 cells (Data not shown) but PHA (10 µg/ml) considerably increased IL-2 production in Molt-4 cells and Propranolol significantly decreased the IL-2 production in PHA stimulated Molt-4 cells after 48 hour of incubation time, compared with untreated control cells dose-dependently (Figure 1 A) (P<0.05).

**Propranolol effect on IL-2 production in human Jurkat** **cells**

IL-2 production was very low in unstimulated human leukemic Jurkat cells (Data not shown) but PHA (10 µg/ml) markedly increased IL-2 production in Jurkat cells and propranolol significantly decreased the IL-2 production in PHA stimulated Jurkat cells after 48 hour of incubation time, compared with untreated control cells dose-dependently (Figure 1 B) (P<0.05).

**II) Propranolol effect on IFN-γ** **production in human leukemic cell lines**

**Propranolol effect on IFN-γ** **production in human Molt-4** **cells**

**Figure 2:**
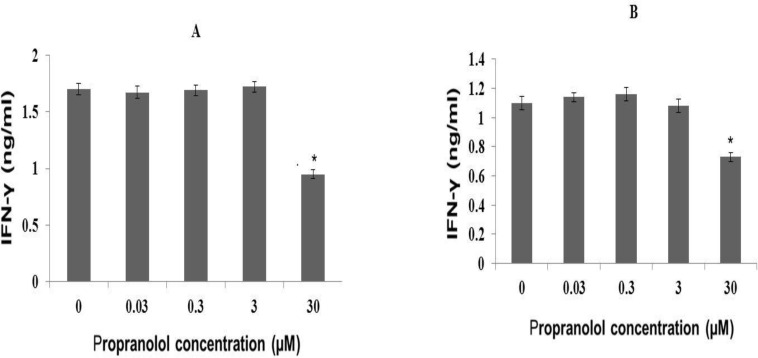
Effect on propranolol on IFN- γ secretion by human leukemic (A) Molt-4 T-cell and (B) Jurkat T-cell lines. The cells (2 × 10 ^6^ cells/ml) were treated with different concentrations of propranolol (0.3-30 μM) for 48 hours in the presence of PHA (10 µg/ml). At the end of treatment, IFN- γ concentration in conditioned medium was measured by ELISA. Data are mean ± SEM of three independent experiments. *P<0.05 was considered significant.

IFN-γ production was rather good in unstimulated human leukemic Molt-4 cells (Data not shown) and PHA (10 µg/ml) markedly increased IFN-γ production in Molt-4 cells. Propranolol significantly decreased the IFN-γ production in PHA- stimulated Molt-4 cells, after 48 hour of incubation time, compared with untreated control cells dose-dependently (Figure 2 A) (P<0.05).

**Propranolol effect on IFN-γ** **production in human Jurkat cells**

IFN-γ production was fairly low in unstimulated human leukemic Jurkat cells (Data not shown), but PHA (10 µg/ml) markedly increased IFN-γ production in Jurkat cells and propranolol significantly decreased the IFN-γ production in PHA- stimulated Jurkat cells, after 48 hour of incubation time, compared with untreated control cells dose-dependently (Figure 2 B) (P<0.05).

## Discussion

 In the present study we showed that propranolol inhibited the PHA-induced secretion of IL-2 and IFN- γ by human leukemic T (Molt-4 and Jurkat) cells. Decrease of IFN-γ by propranolol in our findings is consistent with Seyedi et al. study^17^ in which propranolol significantly diminished IFN-γ secretion in tumor-infiltrating lymphocytes and peripheral blood mononuclear cells (PBMCs) of colorectal cancer patients. Of course it should be noted that in Seyedi et al. study^17^ propranolol decreased IFN-γ secretion at 1µM concentration while in our study propranolol diminished IFN-γ secretion at 30µM concentration and did not show any significant effect at < 30µM concentration. The discrepancy between our results and Seyedi et al. study^17^ might be due to some reasons including kind of cells, number of cells and incubation time intervals used in these studies. In our study, 2×10^6^ cells/well of human leukemic T (Molt-4 and Jurkat) cells were used and measurements were done after 48 h incubation time, whereas Seyedi et al. used 10^5^ cells/well of tumor-infiltrating lymphocytes and PBMCs of colorectal cancer patients and assays were done after 72 hours incubation time. Therefore it may be concluded that different cells have different sensitivity to propranolol or elongation of incubation time make the cells sensitive to much lower concentration of the drug. Seyedi et al. incubated the cells with propranolol 24 hours more than us. Anyway regarding the important role of IFN-γ in inflammation^18^ the anti- inflammatory effect of propranolol reported by other investigators^14^ may be in part due to its suppressive effect on IFN-γ production.

Also, in our study propranolol significantly reduced IL-2 production in human leukemic T cells. As IL-2 has an imperative role in inflammation^19^ the anti- inflammatory effect of propranolol reported by other investigators^14^ may be partially owing to its inhibitory effect on IL-2 production. Reduction of IL-2 by propranolol showed in our study is consistent with Ebbinghaus et al. study^20^ in which propranolol considerably reduced IL-2, IL-17 and transforming growth factor B in supernates of murine lymph nodes and spleen lymphocytes. Moreover our findings are in accordance with Zhou et al. study in which propranolol decreased inflammatory cell and cytokines (tumor necrosis factor-α and interleukin-8) levels in alveolar fluid of rat.^16^ In addition decrease of proinflammatory cytokines such as IL-1β and TNF-α in an experimental model of periodontal disease by propranolol have been shown.^21^ Besides pretreatment with propranolol reversed increase of IL-6 and TNF-α in a mice model. ^22^

According to our previous study,^23^ propranolol did not have any cytotoxic effect on the cell lines examined in this study at the utilized concentrations (0.03-30 μM) and incubation time (48 hours). Hence, the inhibitory effect of propranolol on IL-2 and IFN-γ secretion in the present study is independent of propranolol cell cytotoxicity.

Decrease of inflammatory cytokines (IFN-γ, IL-2) by propranolol in the present study is in accordance with the decrease of vascular endothelial growth factor (VEGF) and matrix metalloproteinase-2 (MMP-2) by propranolol in our previous study ^15^ because VEGF and MMP-2 have important roles in inflammation.^24^^,^^25^

Implication of anti-inflammatory mediators in treatment of ischemic heart failure has been reported.^26^^,^^27^ So, positive effect of propranolol on treatment of many cardiovascular problems such as ischemic heart diseases and heart malfunction ^12^^,^^13^ may be in part owing to its anti-inflammatory effects through inhibition of inflammatory cytokines (IL-2 and IFN-γ) was shown in this study.

It should be considered that in our study, the concentration of propranolol which decreased the IL-2 and IFN-γ production after 48 hours incubation time in vitro was higher than that of usually used in cardiovascular patients. Of course it should be noted that our study was performed in vitro which is different situation from in vivo in patients.

Furthermore, as mentioned before, in Seyedi et al. study^17^ propranolol decreased the IFN-γ and IL-17 production at much lower concentration (1 µM) but higher incubation time (72 hours) than us. As propranolol is used in cardiovascular diseases for long time period,^28^ the drug concentration used in patients might be enough for inhibiting of inflammatory cytokines such as IL-2 and IFN-γ in vivo.

In our study, propranolol reduced IFN-γ and IL-2 (Th1 cytokines) production. As Th1 cells have essential anti-tumor properties,^29^ we might conclude that propranolol decreases anti-cancer immune responses. But antiproliferative and anti-cancer effects of propranolol have been reported by other researchers.^30^^,^^31^ So it seems that anti-tumor effects of propranolol may be mediated by Th1- independent mechanisms. This deduction is consistent with our previous study^15^ in which propranolol reduced VEGF and MMP-2 production, since VEGF and MMP-2 have important roles in tumor progression and metastasis.^32^^,^^33^ Also the important role of some pro-inflammatory cytokines in tumorigenesis has been shown.^34^ Thus the anti-tumor effects of propranolol in some cancers pronounced by other investigators may be in part due to its inhibitory effects on certain pro-inflammatory cytokines.

According to our results propranolol could be considered as a potential inhibitor of pro-inflammatory cytokines and so may have beneficial effects for treatment of inflammatory-based disorders including allergic and autoimmune diseases.

Altogether propranolol as well as its long-lasting use in cardiovascular disorders might be an effective anti-inflammatory agent and have potential implication in management of inflammatory conditions. Further studies about propranolol effects on other inflammatory cytokines/ markers in vitro as well as inflammatory-based diseases in vivo are also needed.

## CONCLUSION

 Propranolol can be considered as a potential inhibitor of pro-inflammatory cytokines and along with its chronic long-term usage in cardiovascular problems could have potential implication in management of inflammatory-based disorders.
